# SOPEZ: study for the optimization of ergonomics in the dental practice - musculoskeletal disorders in dentists and dental assistants: a study protocol

**DOI:** 10.1186/s12995-020-00273-0

**Published:** 2020-07-06

**Authors:** Daniela Ohlendorf, Laura Maltry, Jasmin Hänel, Werner Betz, Christina Erbe, Christian Maurer-Grubinger, Fabian Holzgreve, Eileen M. Wanke, Dörthe Brüggmann, Albert Nienhaus, David A. Groneberg

**Affiliations:** 1 Institute of Occupational Medicine, Social Medicine and Environmental Medicine, Goethe-University, Theodor-Stern-Kai 7, Building 9a, 60596 Frankfurt/Main, Germany; 2 Institute of Dentistry, Department of Dental Radiology, Goethe-University, Frankfurt am Main, Germany; 3 Department of Orthodontics, Medical Center of the Johannes Gutenberg-University Mainz, Mainz, Germany; 4 Competence Center for Epidemiology and Health Services Research for Healthcare Professionals (CVcare), University Medical Center Hamburg-Eppendorf (UKE), Principles of Prevention and Rehabilitation Department (GPR), Institute for Statutory Accident Insurance and Prevention in the Health and Welfare Services (BGW), Hamburg, Germany

**Keywords:** Musculoskeletal disorders, Dentist, Dental assistant, Treatment concept, Dental practice, Nordic questionnaire, Kinematic analysis, RULA, Inertial sensors

## Abstract

**Background:**

Musculoskeletal disorders (MSD) are common among dental professionals. The most common areas affected are the trunk, neck, shoulders and wrists. Current evidence suggests that the causes of MSD can be found in the physical demands of the profession. Posture and movement during treatment is influenced by the arrangement of the treatment concept (patient chair, equipment and cabinets). It has not been investigated whether the ergonomic risk differs between the treatment concepts.

**Methods:**

To evaluate the prevalence of MSD in dental professionals, 1000 responses will be collected from a nationwide (Germany) online questionnaire (mod. Nordic Questionnaire and mod. Meyer questionnaire). In order to assess the ergonomic risk of the treatment techniques used in the four treatment concepts, 3D movement analyses are carried out with inertial sensors. For this purpose, 20 teams of dentists and dental assistants from four dental fields of specializations (generalists, orthodontists, endodontists and oral surgeons) and a student control group will be recruited. Each team will execute field specific standardized treatments at a dummy head. Measurements are carried out in each of the four treatment concepts. The data will be analyzed using the Rapid Upper Limb Assessment (RULA) which will be modified for the evaluation of objective data.

**Conclusions:**

On the basis of these investigations, a substantial gain of knowledge regarding work-related MSD in the field of dentistry and its potential biomechanical causes is possible. For the first time, objective and differentiated comparisons between the four treatment concepts are possible for different fields of dental specialization. Up to now, statically held positions of the trunk and proximal upper extremities, but also the repetitive movements of the hands have been considered a risk for MSD. Since both are included in the RULA, dental activities can be assessed in a detailed but also global manner with regard to ergonomic risks.

## Background

Musculoskeletal disorders (MSD) are common among dental professionals [1–6]. The prevalence of MSD among dentists, dental assistants and dental students varies between 10.8 and 97.9% dependent on the body region according to Lietz et al. [2]. The trunk, neck, shoulders and wrists are the most commonly affected areas [1, 2, 4, 7, 8], often resulting in sick leave or even premature retirement [6, 8–11]. According to Brown et al. [9] a questionnaire survey of early retired dentists from a British insurance company found that MSD are the main cause of ill health retirement at 55%.

In general, the study situation indicates that the causes of MSD are related to the physical demands of the dental profession [3, 12]. The complex and fine-motor activities in the patient’s mouth, which is difficult to inspect, often require the sole attention of the practitioner. Ergonomic posture is therefore neglected in favor of better vision, although initial studies show that an upright trunk posture does not necessarily reduce the quality of treatment [13]. Posture is often static in awkward postures, especially in the trunk, neck and head, while treatment techniques require many, short and repetitive movements of the arms and hands [1, 3].

**Fig. 1:**
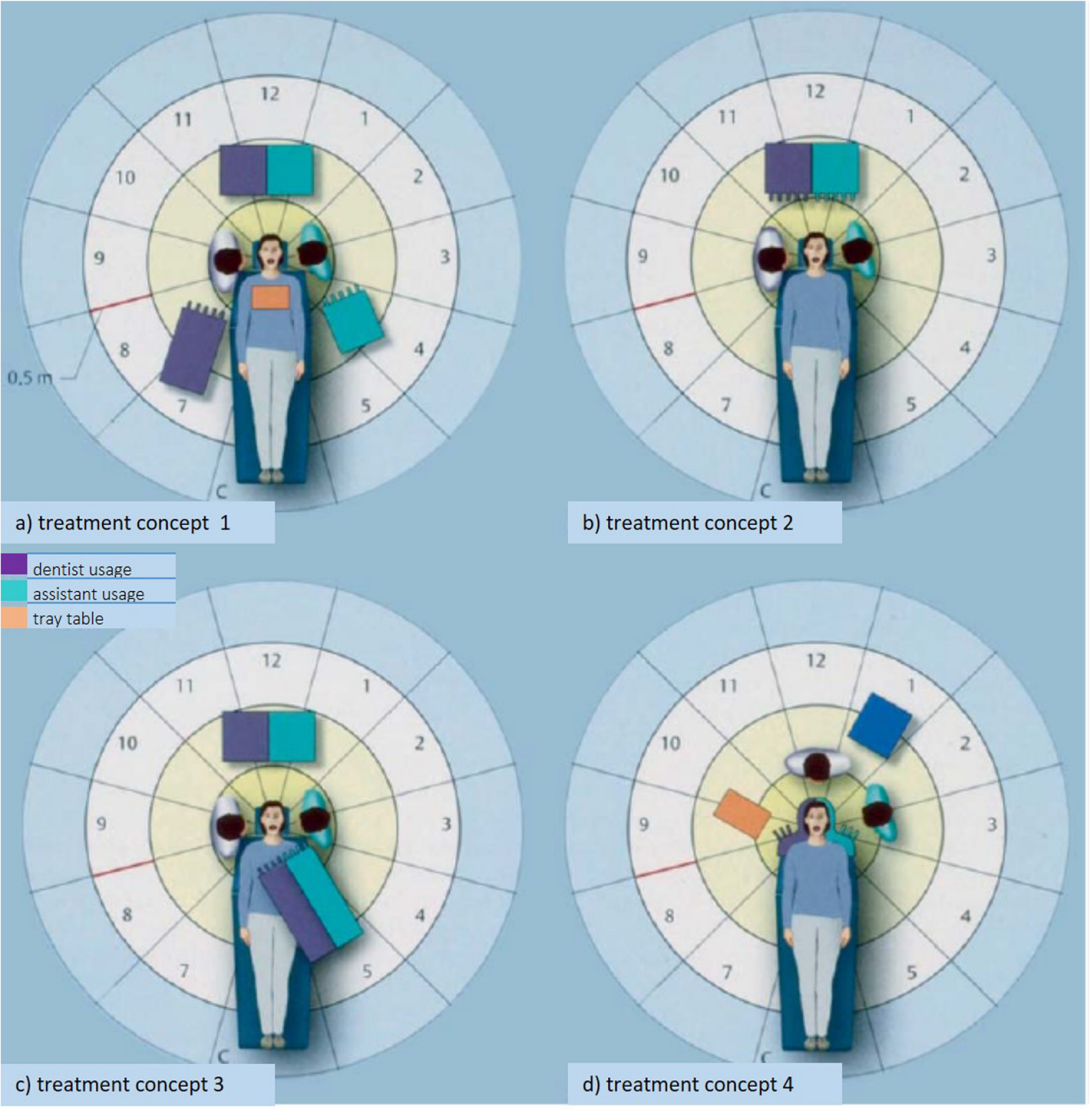
Treatment concepts 1–4 after Kimmel [17]

In addition to the narrow patient’s mouth, the arrangement of the dental workplace (patient chair, equipment and cabinets) also influences the posture and movement of the dental professionals. In the arrangement of the components, a distinction is made between four different treatment concepts [14–16] (Fig. 1). In all four concepts it is planned that the dentist performs treatment together with an assistant. Accordingly, both must have (ergonomically) appropriate access to the necessary equipment and materials and be able to inspect and to work in the patient’s mouth. Although MSD have already been the subject of much research in dentistry, a distinction between the various basic concepts and between the various specializations has not yet been considered.

Current knowledge on MSD in dentistry is mainly derived from questionnaire surveys [1, 5, 6, 8, 18, 19] and from EMG studies [3, 20–22]. In the area of questionnaire surveys on work-related MSD, various approaches have already been carried out [18, 23, 24]. An established tool is the Nordic Questionnaire [25], which has already been used for the survey of musculoskeletal complaints in dentistry [8, 19, 26]. In EMG studies, high average strains were found for the trapezius muscle [20, 22], the splenius capitis muscle [3, 21] and the extensor carpi radialis [22] independent of the treatment performed.

However, objective data from movement analyses on actual posture in dentistry are only available to a limited extent for dentists [12, 27–30], but not for working with assistants. For example Ohlendorf et al. [29, 30], identified static (> 4 s) awkward postures in generalists and orthodontists. These are characterized by flexion and unilateral rotations in the head and trunk area and therefore simulate the typical dental treatment positions. In addition, the same working group was able to show with a combination of task and movement analysis that awkward postures are more likely to occur during dental treatment (generalists: 41% of the working day; orthodontists: 28% of the working day) than during computer work [12, 27]. The subjects worked predominantly in a seated posture (treatment and computer work). The direct comparison of the activities of generalists and orthodontists showed that dentists showed more unfavorable postures [28].

Basically, in the dental fields of specializations there is general dentistry and orthodontics as well as endodontology and oral surgery. It would be possible that the different treatments could influence the posture [3, 7, 12, 27–30]. It is also unclear whether these movement patterns only become established with increasing years of work, or whether students and trainees already acquire unfavorable movement patterns [31]. Overall, there is insufficient information about MSD and the potential influencing factors in dental professions to recommend appropriate preventive measures for dentists and their assistants.

The Rapid Upper Limb Assessment (RULA) [32, 33] is frequently used internationally to assess the ergonomic risk of workflow processes. The focus is on the body regions neck, shoulders, trunk, arms and hands with a predominantly kinematic approach. Although RULA was originally developed for observational assessment, there are already approaches to apply the tool to data from movement analyses collected with inertial sensors [34–36].

Various approaches are available to adequately respond to any possible ergonomic hazards. While changes in the arrangement of the equipment in the four treatment concepts can be assigned to the area of relational prevention, there are already behavioral prevention approaches. For example, ergonomic training courses on posture and treatment techniques are offered commercially. However, their effectiveness has hardly been scientifically investigated to date. Besides the education of ergonomic working methods, strength training can also be a promising approach to behavioral prevention in dentistry. Strength training is intended to strengthen the trunk muscles so that awkward postures can also be compensated more effectively [37]. Similar to ergonomic training, there are no studies to date on the effectiveness of such strength training specifically for dentists.

### Aims

The aim of this study is to comprehensively investigate MSD in the dental professional field. Initially, a Germany-wide online questionnaire will be used to collect data on MSD, work flow and health-related activities among dental professionals. Furthermore, an ergonomic risk assessment of the cooperative treatment of dentists and dental assistants based on objective movement data will be carried out for the first time. This will be done for four specializations (generalists, oral surgeons, endodontists and orthodontists) and a control group of dental students and dental assistant trainees at each of the four treatment concepts. The activities will be carried out in a standardized manner on the dummy head, so that differences between the treatment concepts can be recognized.

## Methods

### Subjects

#### Online survey

For the Germany-wide online survey, a total of at least 1000 completed questionnaires are targeted in the return. The target group includes dentists and dental assistants as well as dental students and dental assistant trainees.

#### Biomechanical analysis

For the biomechanical measurements, the aim is to recruit 20 teams of dentists and dental assistants in each of the four fields of dental specialization (generalists, endodontists, oral surgeons and orthodontists) and also in the control group (students/trainees). Both women and men between the ages of 18–65 years who treat right-handed are included.

Exclusion criteria include current injuries to the musculoskeletal system (e.g. herniated discs, spinal injuries), rheumatic diseases, severely restrictive malformations (scoliosis) of the spine or stiffened spinal joints (pathological or surgical), genetically determined muscular diseases and surgery less than 2 years ago. In the control group, dental students and trainees as dental assistants with treatment experience are included.

The study is approved by the Ethics committee of the Department of Medicine of the Goethe University Frankfurt am Main (356/17).

### Recruitment

The distribution of the questionnaire link as well as the search for participants for the biomechanical measurements will be carried out via the different German dental associations. In order to reach the highest possible number of practicing dentists and dental assistants for the questionnaire, the acquisition of study participants will be done through the state dental associations of all German federal states. In addition, a flyer for the survey is to be created, which will be distributed at various dental meetings. In order to include a high number of dental students, all medical university clinics in Germany will be contacted.

### Measurement protocol

#### Online survey

The online questionnaire is composed and supplemented from the Nordic Questionnaire by Kuorinka et al. [25] and from the questionnaire according to Meyer [18] on the workload of dentists working in a private practice.

Questions from the Nordic Questionnaire [25] on the prevalence of MSD in the regions of neck, upper and lower back, shoulders, elbows and wrists/hands as well as hips/thighs, knees and ankles/feet are included in the questionnaire. This is done for lifetime prevalence, 12-month prevalence, 3-month prevalence and 7-day prevalence. From Meyer’s questionnaire [18] questions on work restrictions, personal/psychological occupational stress and activities of daily living (ADLs) are included. Questions on health-related activities are also included, for example, whether the person has already participated in an ergonomics training course. In addition, working habits and spatial conditions of daily work as well as previous illnesses of the musculoskeletal system are also examined. Sociodemographic data and medication intake are also included.

The questionnaire will be created online via the SoSci Survey server. A pretest will be carried out to check the practicability and quality of the questionnaire. In the pretest, in addition to the regular answering of the questionnaire, comments on the individual pages should be possible in order to note remarks and difficulties in understanding. Based on the pretest the questionnaire will be adapted if necessary. The questionnaire is used in this study both in the Germany-wide survey and prior to the biomechanical analysis.

#### Motion capturing

The recording of the posture in the different test situations will be carried out by means of the inertial motion capture system MVN Link from XSens (Enschede, Netherlands). Sampling rate is 240 Hz and the measurement error is specified by the manufacturer as ±1%. By means of 17 sensors 22 joints will be interpolated.

**Fig. 2:**
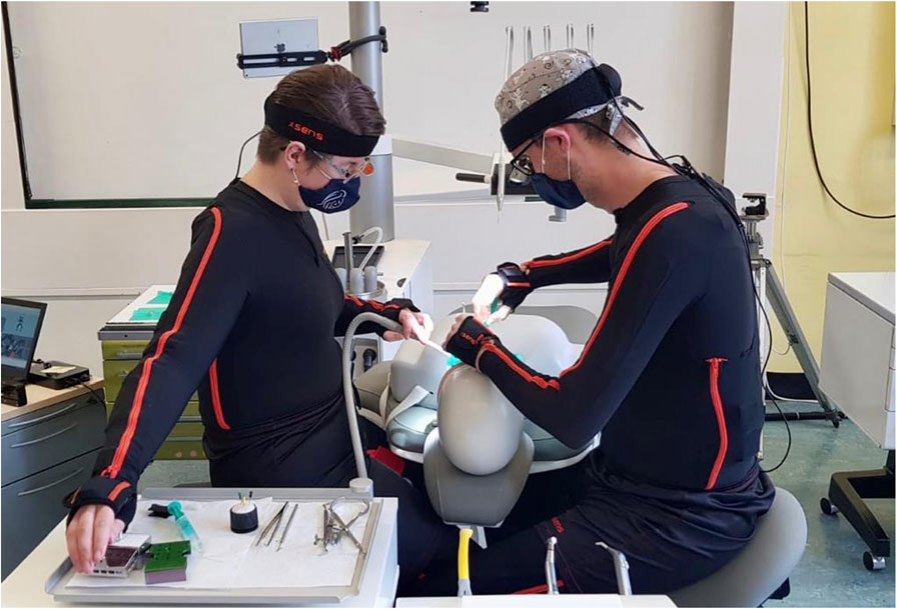
Treatment of the phantom head when wearing the MVN Link system by Xsens. Subjects can move freely without interference with the measurement system

After the measuring suit has been put on, the recordings take place over a period of 1 h on each of the four treatment concepts (4 h in total): On a dummy head, which is attached to each of the four treatment concepts, dental activities per specialization are carried in a standardized sequence (Fig. 2). The activities within each specialization are performed in all four quadrants (Table 1 for treatments of the respective fields of dental specialization). In addition to the individual biomechanical analysis, the interaction between dentist and dental assistant is recorded synchronously. In order to be able to allocate the activities exactly, the entire measurement sequence is filmed simultaneously in a total view (iPad Air, Apple Inc., Cupertino, California, United States; resolution: 1080p HD, 120fps). Therefore, measurement system and the camera will be synchronized by means of the software. Due to the character of the work, no randomization of the tasks will be carried out.

**Table 1:** Standardized tasks for the fields of specialization: The group of general dentists and the control group of students execute the same tasks. All tasks in the four dental quadrants will be executed on each of the four treatment concepts

	Task	Quadrant 1	Quadrant 2	Quadrant 3	Quadrant 4
**General Dentistry/Students**		**Tooth filling of tooth 16**	**Preparation of tooth 26 for crown uptake**	**Root canal treatment on tooth 35**	**Tartar removal in the 4th quadrant**
	**1**	prepare tooth cavity with a cylindrical diamond bur and the use of wedges	Occlusal reduction using an occlusal reducer	Performing an entrance cavity and trepanation on tooth 35 using a diamond-coated cylinder	Removal of supra- and subgingival tartar/calculus using scalers an curettes
	**2**	Create a Tofflemire die using a die clamp	chamfer preparation using a torpedo-shaped diamond burand approximal reducer	Find the channel entrance using an endo file	
	**3**	Tooth filling with ketac® while using a ketac®-set and a cougar/heidemann		Manual preparation of the canal using an ISO 20-40 endo file with regular irrigation using a irrigation cannula	
**Endodontology**		**Root canal treatment of tooth 16**	**Root canal treatment of tooth 26**	**Root canal treatment of tooth 36**	**Root canal treatment of tooth 46**
	**1**	Application of the rubber dam	Application of the rubber dam	Application of the rubber dam	Application of the rubber dam
	**2**	Trepanation of the tooth and access preparation including the enlarging of the root canal entrance	Trepanation of the tooth and access preparation including the enlarging of the root canal entrance	Trepanation of the tooth and access preparation including the enlarging of the root canal entrance	Trepanation of the tooth and access preparation including the enlarging of the root canal entrance
	**3**	Root canal preparation with hand files at a certain working length, irrigation after each file and remove of the rubber dam	Root canal preparation with hand files at a certain working length, irrigation after each file and remove of the rubber dam	Root canal preparation with hand files at a certain working length, irrigation after each file and remove of the rubber dam	Root canal preparation with hand files at a certain working length, irrigation after each file and remove of the rubber dam
**Orthodontics**		**Multiband Treatment**	**Multiband Treatment**	**Multiband Treatment**	**Multiband Treatment**
	**1**	Acid etching	Acid etching	Acid etching	Acid etching
	**2**	direct bonding of braces on teeth 1, 3, 4 and 6 and opening of self-ligating braces	direct bonding of braces on teeth 1, 3, 4 and 6 and opening of self-ligating braces	direct bonding of braces on teeth 1, 3, 4 and 6 and opening of self-ligating braces	direct bonding of braces on teeth 1, 3, 4 and 6 and opening of self-ligating braces
	**3**	insertion of archwire	Insertion of archwire	Insertion of archwire	Insertion of archwire
	**4**	Integration of brackets 3 using elastic ligation	Integration of brackets 3 using elastic ligation	Integration of brackets 3 using elastic ligation	Integration of brackets 3 using elastic ligation
	**5**	Integration of brackets 1 and 4 using metal ligation	Integration of brackets 1 and 4 using metal ligation	Integration of brackets 1 and 4 using metal ligation	Integration of brackets 1 and 4 using metal ligation
	**6**	Debonding of brackets	Debonding of brackets	Debonding of brackets	Debonding of brackets
**Oral surgery**		**Surgical removal of tooth 13**	**Surgical removal of tooth 23**	**Surgical removal of tooth 38**	**Surgical removal of tooth 48**
	**1**	palatinal and marginal incision in regio 16 to 11.	Vestibular and marginal incision in regio 21 to 25.	Crestal incision in regio 38 with a mesial relieving incision.	Crestal incision in regio 48 to 44.
	**2**	Exposure of the palatinal impacted tooth 13 by osteotomy using a surgical round bur. If necessary, cut through the tooth using a Lindemann bur. Remove the tooth 13 using a Bein root elevator or a dental forceps. Curretage of the dental sac.	Exposure of the vestibular impacted tooth 23 by osteotomy using a surgical round bur. If necessary, cut through the tooth using a Lindemann bur. Removal of the tooth 23 using a Bein root elevator or a dental forceps. Curretage of the dental sac.	Exposure of the impacted tooth 38 by osteotomy using a surgical round bur. Removal of the tooth 38 using a Bein root elevator or a dental forceps. Curretage of the dental sac.	Exposure of the impacted tooth 48 by osteotomy using a surgical round bur. If necessary, cutting through the tooth using a Lindemann bur. Removal of the tooth 48 using a Bein root elevator or a dental forceps. Curretage of the dental sac.
	**3**	Wound closure with single loop interrupted sutures	Wound closure with single loop interrupted sutures.	Wound closure with single loop interrupted sutures.	Wound closure with single loop interrupted sutures.

### Evaluation criteria

The endpoints of the questionnaire are mainly collected through selection options (nominal scales) or ordinal scaled items. In some cases, open answers are also possible.

From the data of the biomechanical analysis, a risk score is determined for every recorded frame using the Rapid Upper Limb Assessment (RULA) [38]. On the basis of this amean score and SD for wrist and arm (Section A) and neck, trunk and leg (Section B) can be determined via RULA [38]. Furthermore, the percentage of time spent in the respective risk scores and at high risk should be determined. Both score values result in an overall score that indicates the level of MSD risk of the examined activity [38].

### Statistical data analysis

Only those questionnaires that have been fully completed will be included in the questionnaire evaluation. The data formatting is done via Excel. The questionnaires are checked for plausibility in the answering behavior. A category system will be developed for open answers. The questionnaire will be evaluated in IBM SPSS Statistics 26, and the questionnaire data will be evaluated descriptively, separately for dentists and dental students, and for dental assistants and trainees. Therefore, measures of location and dispersion corresponding to the scale level are calculated.

The data analysis of the biomechanical measurements is performed with MATLAB® vR2018a software (The Mathworks Inc., Natick, MA, USA). The RULA risk scores will be presented separately for dentists and dental assistants in each of the four treatment concepts for each field of dental specialization.

## Discussion

The aim of this study, is to assess the prevalence of MSD in dental professionals for all relevant regions of the body. In addition, a kinematic movement analysis will be used to record typical dental work procedures on all four treatment concepts. With the use of the RULA, the postures recorded will be examined with regard to their ergonomic risk. On the basis of these investigations, a comprehensive knowledge gain regarding work-related MSD in dentistry and their possible biomechanical causes is possible. Such an approach has not yet been published.

The current evidence in the field of task and movement analyses indicates that dental work is largely characterized by unfavorable static postures of the neck, back and shoulders [20, 29, 39]. Such postures are not only in dentistry, but also generally among the risk factors for occupational MSD [40]. To maintain these static postures, a permanent isometric contraction of > 50% of the body’s muscles is necessary [39]. This can lead to changes in blood flow, contractile activity and, in the long term, morphology and nociception [20]. A kinematic 3D analysis of dental activity by Finsen et al. [3] from 1999 (recordings from two video cameras) shows that a neck flexion of > 30° was held for 82% of working time. An arm abduction > 30° was recorded for 36% of the working time. In order to obtain even more detailed movement analyses of the whole body during dental work, the newly developed inertial sensors used here are preferable, as the practitioners are positioned close to the patient’s chair and with their legs even below it. The legs of dentist and assistant are often interlocked and the patient chair blocks the cranial view on the legs. When data is collected using optical motion capture, the field of view of the cameras could therefore be impaired, making it difficult to take pictures of the entire body.

The ergonomic evaluation of the inertially collected data using the RULA allows a detailed ergonomic risk assessment of the work processes. While observational methods are traditionally filled in manually and therefore can only provide a rather subjective snapshot, the approach presented allows an objective risk assessment for each frame captured. The continuous data therefore contribute to the breakdown and ergonomic evaluation of smallest work sections [34, 36]. On the basis of this, a better understanding of MSD risks in dentistry can be contributed.

A further advantage of the RULA is that both the statically held positions of the trunk and the proximal upper extremities are taken into account, as well as the repetitive movements of the hands, which also poses a risk for MSD [39, 41]. Questionnaire surveys in dental professionals have already shown in the past that wrists and hands are also affected by MSD [1, 39]. In addition to the movements, the vibration of the devices also contributes to this [42]. In Germany, only carpal tunnel syndrome is acknowledged as a musculoskeletal occupational disease in dentistry [43].

Although the body of literature is already relatively large with regard to ergonomic risk in dentists, a comparison of the different treatment concepts is still missing. Thus, it has not yet been considered whether the arrangement of the inventory and the positioning of dentist and assistant in relation to the patient have an influence on posture and movement sequences. If differences occur here, this information can be integrated into the development of preventive measures.

The aim should be that both practitioners complement each other in their grasping movements to the instruments and in their work in the patient’s mouth. This should reduce the physical strain on both. The presented study design with the standardization of the tasks performed on a dummy head allows comparisons between the treatment concepts for the first time.

Furthermore, the comparison of the specialization fields with the control group can be expected to contribute to the clarification of the question at which stage of the work experience unfavorable postures occur. Finsen et al. [3] for example, were able to show by means of a questionnaire survey that the prevalence of MSD increases with increasing years of work experience.

### Limitations

The questionnaires will be distributed through a non-probabilistic procedure and the people contacted can decide to participate self-selectively. Therefore, a distortion is possible, as especially persons who are interested in ergonomic topics in dentistry could be motivated to participate. The representativeness of the results could be affected.

Since the measurements are carried out in laboratory settings, it is possible that no realistic measurement conditions are available. The recordings are not made in the dentists own practice, so that routine work processes may not be correctly recorded. In addition, a dummy head is used to improve the standardization of work processes. The movement frame of the dummy head is based on physiological conditions. The risk of setting unrealistic head positions on the dummy is therefore low. However, under real conditions, it should be noted that the mobility of the cervical spine varies in patients.

No conclusions can be drawn on the dose-response relationship between posture and MSD on the basis of this study. If, however, it is known which basic concepts and working methods are fundamentally in favor of unergonomic working methods, follow-up studies in this field can specifically determine the duration and frequency of treatments in practice.

### Future research

Based on the data to be collected, behavioral preventive interventions can also be planned, such as an ergonomic training or a strength training. While an ergonomic training aims to establish less risky postures, strength training aims to strengthen muscular resources in order to increase resilience. Both approaches could have a positive effect on work behavior and thus on the prevalence of MSD. However, ergonomic training has hardly been researched so far. Only in a pilot study, a 1.5 h ergonomics training with dental students using an electronic posture trainer was evaluated [13]. After the ergonomics training, the students were supposed to check each other’s posture and after 1 week the post-test took place, in which the test persons had adapted a more upright posture, which was not at the expense of the preparation quality, which was also evaluated [13]. However, it is questionable whether this posture can be maintained in the long term. The effectiveness of strength training in reducing pain in chronic lower back pain [44–48] and neck/shoulder pain [49–51] has already been demonstrated in numerous studies. It can be assumed that this can also help dentists and dental assistants to reduce MSD. Furthermore, the method established here for assessing ergonomic risk offers new possibilities for testing the effectiveness of interventions. Thus, an intervention-dependent change in working posture can be made visible. However, there is currently no evidence of this.

## Conclusion

The aim of this study is, to contribute information on the prevalence of MSD in dental professionals and on the ergonomic risk potential of treatment procedures, focusing on the four different treatment concepts. From the perspective of prevention, there is great potential in avoiding work-related MSD. In this study, the approach is to investigate the treatment methods in dentistry. The aim is to provide both dentists and dental assistants with information on ergonomic working methods.

## Data Availability

Not applicable.

## Abbreviations

MSD: Musculoskeletal disorders
p: Pixel
fps: Frames per second
